# Reversible Control of Nanoparticle Functionalization and Physicochemical Properties by Dynamic Covalent Exchange**

**DOI:** 10.1002/anie.201409602

**Published:** 2015-01-14

**Authors:** Flavio della Sala, Euan R Kay

**Affiliations:** EaStCHEM School of Chemistry, University of St Andrews North Haugh St Andrews KY16 9ST (UK)

**Keywords:** dynamic covalent chemistry, gold nanoparticles, hydrazones, supramolecular chemistry

## Abstract

Existing methods for the covalent functionalization of nanoparticles rely on kinetically controlled reactions, and largely lack the sophistication of the preeminent oligonucleotide-based noncovalent strategies. Here we report the application of dynamic covalent chemistry for the reversible modification of nanoparticle (NP) surface functionality, combining the benefits of non-biomolecular covalent chemistry with the favorable features of equilibrium processes. A homogeneous monolayer of nanoparticle-bound hydrazones can undergo quantitative dynamic covalent exchange. The pseudomolecular nature of the NP system allows for the in situ characterization of surface-bound species, and real-time tracking of the exchange reactions. Furthermore, dynamic covalent exchange offers a simple approach for reversibly switching—and subtly tuning—NP properties such as solvophilicity.

Despite tremendous advances in the preparation of nanoparticles (NPs) from a range of materials,^1^ manipulation and characterization of NP surface functionality remains a crucial challenge in the quest to exploit the often remarkable properties observed within this newfound region of chemical space. Direct incorporation of surface-bound functional molecules during NP synthesis is intrinsically restrictive, demanding compatibility with the synthesis conditions. Postsynthetic substitution of temporary surface species in a “ligand exchange” process can facilitate the introduction of a wider range of surface-bound functionalities, independent of the NP synthesis methods.^2^ Yet, such processes are often irreversible, inefficient, and can lead to NP surface reconstruction or etching.

A generalizable synthetic approach whereby a set of NP “building blocks” may be predictably functionalized, manipulated, and assembled is therefore highly desirable. The potential of such a concept is well exemplified by oligonucleotide-functionalized nanomaterials.^3^ Yet, biomolecular methods only operate within tightly defined conditions and offer limited scope for customization. On the other hand, non-biomolecular strategies will allow the full gamut of synthetic chemistry to be exploited in the optimization of nanomaterial structure, function, and properties. Innovative designs exploiting noncovalent interactions for NP functionalization,^4^ aggregation,^5^ and surface immobilization^6^ have recently been explored, but these non-biomolecular systems cannot yet match the stability, specificity, and selectivity of oligonucleotide hybridization. Postsynthetic covalent modification of NP-bound monolayers is an attractive alternative, but traditionally this strategy has relied on kinetically controlled reactions,^7^ which at best produce statistical mixtures of products and only offer one-shot opportunities for functionalization. Thermodynamically controlled covalent bond-forming reactions instead combine the error-correcting and stimuli-responsive features of equilibrium processes with the stability and structural diversity of covalent chemistry.^8^ The first examples of dynamic covalent exchange taking place on 2D surface-confined molecular monolayers,9a–c or at the surface of self-assembled phospholipid bilayers,9d,e have recently been reported. We now show that such reactions may also be successfully performed on 3D NP-bound monolayers. We present prototypical “dynamic covalent NP building blocks”: gold nanoparticles (AuNPs) bearing a homogeneous monolayer of *N*-aroylhydrazones (Figure 1), through which reversible control of NP functionalization and properties can be achieved.

**Figure 1:**
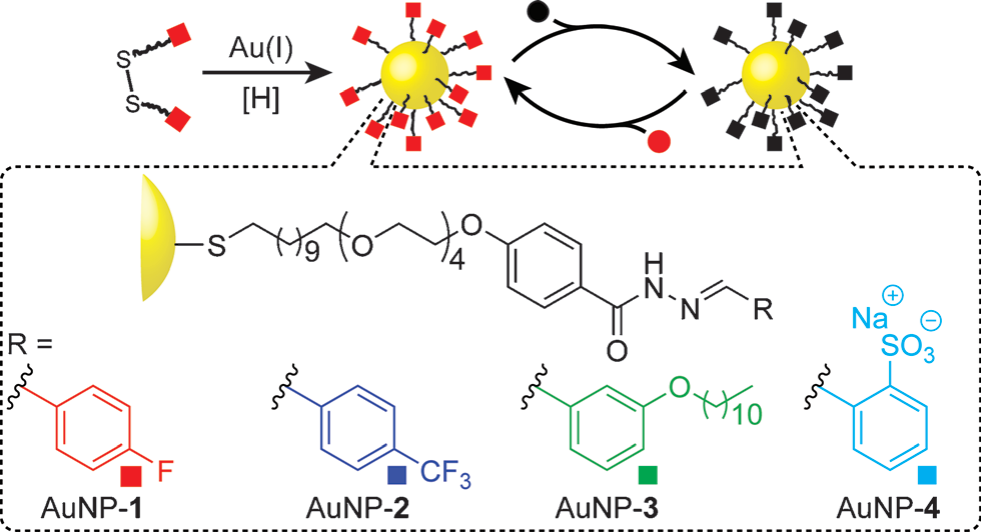
Preparation and reversible surface modification of a dynamic covalent NP building block exploiting *N*-aroylhydrazone surface monolayers.

Hydrazones display stability under a wide range of conditions, yet undergo covalent exchange reactions in the presence of acid or nucleophile catalysts,^10^ making them particularly useful for creating dynamic covalent systems with good differentiation between kinetically labile and locked states.^11^ This combination of behaviors likewise appeared ideal for a robust but exchangeable linkage for the construction of dynamic covalent AuNPs.^12^ Ligand **1** bears an *N*-aroyl hydrazone unit, connected through an aliphatic linker to a thiolate functionality for binding to AuNP surfaces (Figure 2 a).^13^ The alkyl linker encourages the formation of a well-ordered surface monolayer, maximizing van der Waals interactions between neighboring chains,2a whereas the outer tetraethylene glycol unit confers compatibility with polar solvents and conformational flexibility at the dynamic covalent reactive site.^14^

**Figure 2:**
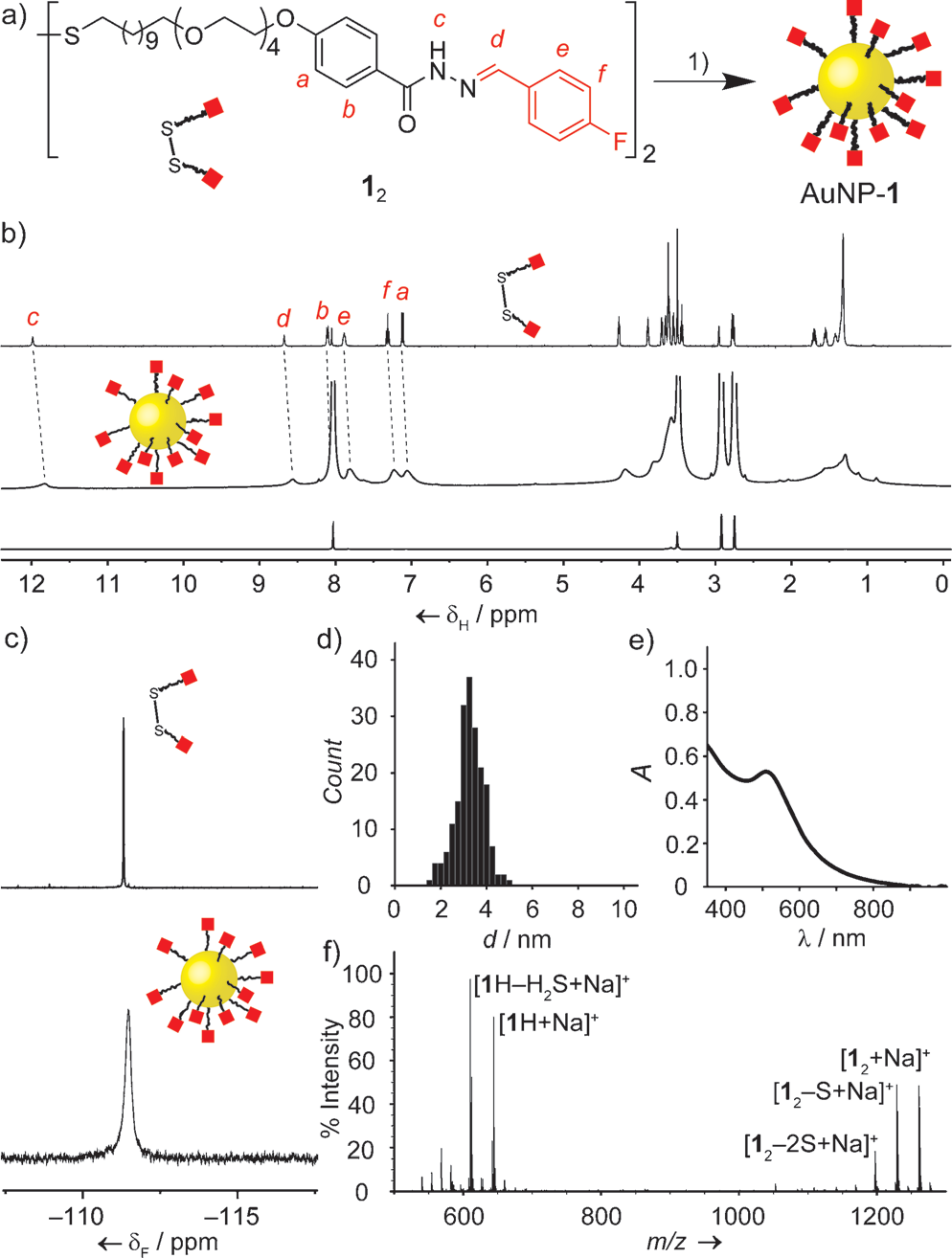
Synthesis and characterization of AuNP-1. a) Nanoparticle synthesis. 1) AuPPh_3_Cl, borane *tert*-butylamine complex, DMF/THF 1:9, RT, 6 h. b) ^1^H NMR spectra ([D_7_]DMF, 500.1 MHz, 295 K): 1_2_ (top); AuNP-1 (middle); AuNP-1­ *T*_2_-filtered spectrum (bottom). Signals at 8.02, 3.50, 2.92, and 2.75 ppm correspond to residual nondeuterated solvent and water. c) ^19^F NMR spectra ([D_7_]DMF, 470.5 MHz, 295 K): 1_2_ (top); AuNP-1 (bottom). d) Size distribution of a representative batch of AuNP-1 (mean diameter 3.39±0.61 nm). e) UV/Vis spectrum of AuNP-1 in DMF (SPR *λ*_max_=509 nm). f) LDI-MS of AuNP-1.

Gold nanoparticles bearing a homogeneous monolayer of **1** were prepared in a one-step, single-phase process,^15^ which consistently yielded NPs of mean diameters in the range of 2.8–3.4 nm, with dispersities <20 % (Figures 2 d and S6), and exhibiting a well-defined surface plasmon resonance (SPR, Figures 2 e and S6). The absence of surfactants or temporary ligands facilitated the preparation of single-component monolayers, while all unbound contaminants could be removed by NP precipitation and washing. Verification of both comprehensive purification, and the structural integrity of NP-bound **1**,^16^ were essential for being able to unambiguously characterize the surface-confined dynamic covalent processes. AuNP-**1** displays characteristically broad ^1^H and ^19^F NMR spectra (Figure 2 b and c) consisting only of the resonances expected for a single-component monolayer of **1**. The absence of nonsolvent unbound contaminants was confirmed by *T*_2_-filtered ^1^H NMR spectroscopy using the recently developed CPMG-*z* pulse sequence (Figures 2 b, bottom, and S3).^17^ Corroboration of the surface-bound molecular structure was provided by LDI-MS, whereby all major ions could be assigned as originating from desorbed **1** (Figures 2 f and S4).^18^ Finally, only products consistent with a homogeneous monolayer of **1** were detected after iodine-induced oxidative ligand stripping from AuNP-**1** (Figure S5).

Directly tracking reactions that occur on molecules confined to non-uniform faceted surfaces, within a heterogeneous population of NPs, presents several challenges. Inherently low concentrations, fast transverse relaxation, and chemical shift heterogeneity combine to yield broad, weak ^1^H NMR spectra for NP-bound molecules (Figure 2 b, middle), making quantitative deconvolution of resonances from structurally similar species extremely challenging.^19^ Incorporating fluorine labels allowed us to exploit the significant chemical shift dispersion and excellent sensitivity of ^19^F NMR spectroscopy to interrogate the composition of hydrazone-bound monolayers before and after dynamic covalent exchange reactions (Figure 3).

**Figure 3:**
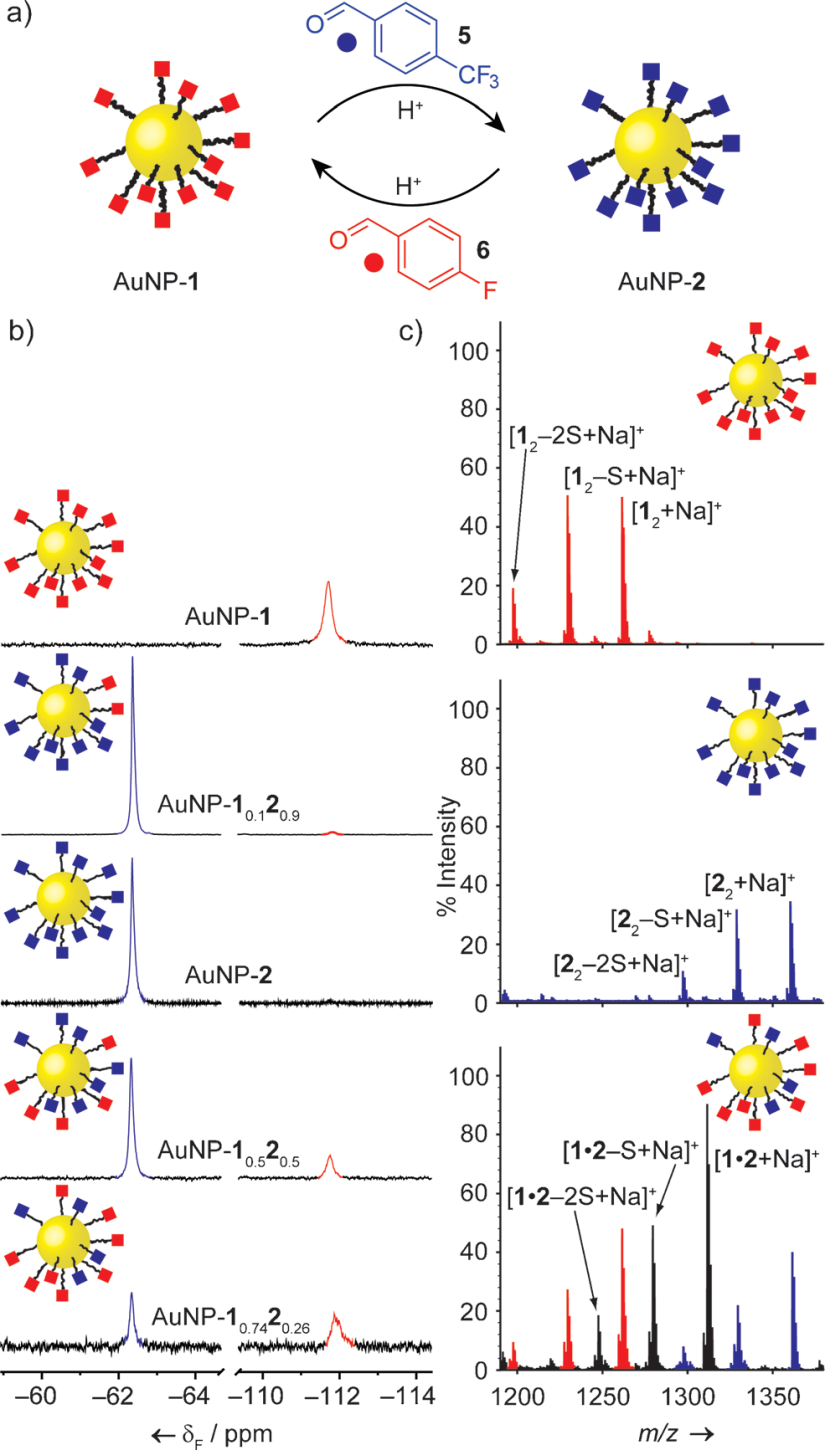
a) Hydrazone exchange between AuNP-1 and AuNP-2. Conditions: aldehyde (20 equiv with respect to 1), CF_3_CO_2_H (5 equiv with respect to 1), D_2_O/[D_7_]DMF 1:9, 50 °C. b) Partial ^19^F NMR spectra ([D_7_]DMF, 470.5 MHz, 295 K), from top to bottom: AuNP-1; AuNP-1_0.1_2_0.9_; AuNP-2; 1_0.5_2_0.5_; AuNP-1_0.74_2_0.26_ . c) Partial LDI-MS spectra of AuNP-1 (top), AuNP-2 (middle), and AuNP-1_0.74_2_0.26_ (bottom).

A stable colloidal suspension of AuNP-**1** in 10 % D_2_O/[D_7_]DMF was treated with an excess of *p*-(trifluoromethyl)benzaldehyde (**5**) and CF_3_CO_2_H.^20^ After 16 h at 50 °C, ^19^F NMR spectroscopy showed that the signal for NP-bound *p*-fluorobenzylidene hydrazone **1** had decreased in intensity and two new resonances had appeared: one corresponding to free *p*-fluorobenzaldehyde (**6**), and another corresponding to NP-bound *p*-(trifluoromethyl)benzylidene hydrazone **2** (see Figure S10 for full sweep width crude and purified spectra). Unbound molecular species (released **6**, excess **5**, and CF_3_CO_2_H) were removed by NP precipitation and washing with nonsolvents, yielding a NP sample with a mixed monolayer comprising 90 % hydrazone **2** and 10 % hydrazone **1** (AuNP-**1**_0.1_**2**_0.9_, Figure 3 b). By subjecting this sample again to the same exchange conditions, followed by purification as before, yielded a pure sample of AuNP-**2** (Figure 3 b). A homogeneous monolayer of **2** was confirmed by ^19^F and ^1^H NMR spectroscopy (Figures 3 b, middle, S9, and S11), LDI-MS (Figures 3 c, middle, and S12), and oxidative ligand stripping (Figure S13).^21^

The dynamic covalent exchange process is entirely reversible. Treatment of AuNP-**2** with **6**, under identical exchange conditions to before, produced a sample displaying a mixed monolayer of the two hydrazones in the ratio 1:1 (AuNP-**1**_0.5_**2**_0.5_, Figure 3 b). Subjecting this sample to a further excess of **6** increased the ratio of hydrazones **1**:**2** in the monolayer to approximately 3:1 (AuNP-**1**_0.74_**2**_0.26_, Figure 3 b). Peaks corresponding to mixed disulfide in the LDI-MS of AuNP-**1**_0.74_**2**_0.26_ (Figure 3 c, bottom) indicate the intimate mixing of hydrazones on the NP surface,^18^ whereas the lower extent of exchange in this reverse process is in line with the greater stability expected for the *p*-(trifluoromethyl)benzylidene hydrazone.10c Importantly, these mixed hydrazone samples allowed us to confirm that quantification of the monolayer composition by integrating the broad NP-bound ^19^F NMR signals was consistent with the results of iodine-induced oxidative ligand stripping and subsequent analysis of the released molecular species (Figure S17).

The ability to quantify both NP-bound and unbound species using ^19^F NMR spectroscopy allowed us to track hydrazone exchange in real time and explore the effects of surface confinement on reactivity. The concentrations of all four fluorinated species (AuNP-**1**, AuNP-**2**, **5**, **6**) were monitored during the exchange of AuNP-**1** with aldehyde **5**.^22^ Comparing the resulting kinetic profile to that of a freely dissolved model compound under the same conditions (Figure S20) indicates a clear kinetic inhibition for the NP-bound reaction. Fitting to derive apparent rate constants (Table S1),^22^ counterintuitively revealed the inhibitory effect to be stronger in one reaction direction (*k*_NP_/*k*_MOL_(F→CF_3_)=0.2) than the other (*k*_NP_/*k*_MOL_(CF_3_→F)=0.5), corresponding to an equilibrium endpoint that favors AuNP-**1** more strongly than predicted by the model reaction in bulk solution. Slower kinetics for the NP-bound process might be expected on the basis of simple steric arguments. However, it is unclear whether the very small increase in size on converting **1** to **2** can explain the differential effect on the exchange rates, or whether other intra-monolayer interactions or local concentration effects are also at play.

Mild and reversible methods for postsynthetic NP modification are highly desirable and would have significant benefits for nanomaterial property control, handling, and processability. For example, tuning solvent compatibility is often required to match an optimized NP synthesis route with a specific end application,^23^ yet existing methods involve either encasing a nanoconstruct in a polymeric modifier, encapsulation in micelles, or completely replacing the surface ligands. The latter strategy may be considered as a dynamic exchange of the Au—S bond.^2^ However, completely replacing the stabilizing monolayer is a relatively harsh and slow process. Whereas hydrazone exchange at 50 °C (as described in Figure 3) reaches 90 % exchange within 24 h, the ligand exchange of AuNP-**1** with disulfide **2**_2_ takes several days to reach an endpoint exhibiting far lower conversion (63 %) under analogous conditions (Figure S21), and does not proceed at all at ambient temperatures.^24^ By exchanging only simple units on the periphery of the stabilizing monolayer, dynamic covalent exchange occurs rapidly under mild conditions; it furthermore avoids the necessity for multistep synthesis of several thiol-containing ligands, offers simple purification of the modified NPs from the molecular exchange species, and is entirely reversible.^24^

To demonstrate the potential of dynamic covalent exchange for reversible property control, we sought to introduce simple aldehyde exchange units, chosen to confer different solvophilic characteristics on our dynamic covalent AuNP building blocks (Figure 4). AuNP-**1** functionalized with *p*-fluorobenzylidene hydrazone showed good colloidal stability only in polar aprotic solvents such as DMF and DMSO (Figure 4, top). Treating AuNP-**1** with an excess of hydrophobic aldehyde **7** and CF_3_CO_2_H in 10 % D_2_O/[D_7_]DMF at 50 °C resulted in complete precipitation of the NPs within 1.5 h. The solid was easily recovered by centrifugation, and then purified from all molecular species by redispersion in methanol followed by precipitation with hexane. The resulting residue exhibited markedly different solubility properties to AuNP-**1** and could be readily re-redispersed in organic solvents of intermediate polarity, such as chloroform or tetrahydrofuran (AuNP-**3**, Figure 4, left). Analysis of the reaction supernatant by ^19^F NMR spectroscopy indicated >95 % conversion of the starting NP-bound *p*-fluorobenzylidene hydrazones (Figure S23). On the other hand, ^1^H NMR and LDI-MS analysis of the new NP sample (Figures S22 and S24) were consistent with the expected *m*-alkoxybenzylidene hydrazone. Clearly, dynamic covalent exchange of NP-bound hydrazones occurred to give AuNP-**3** and the consequent marked change in solvent compatibility.

**Figure 4:**
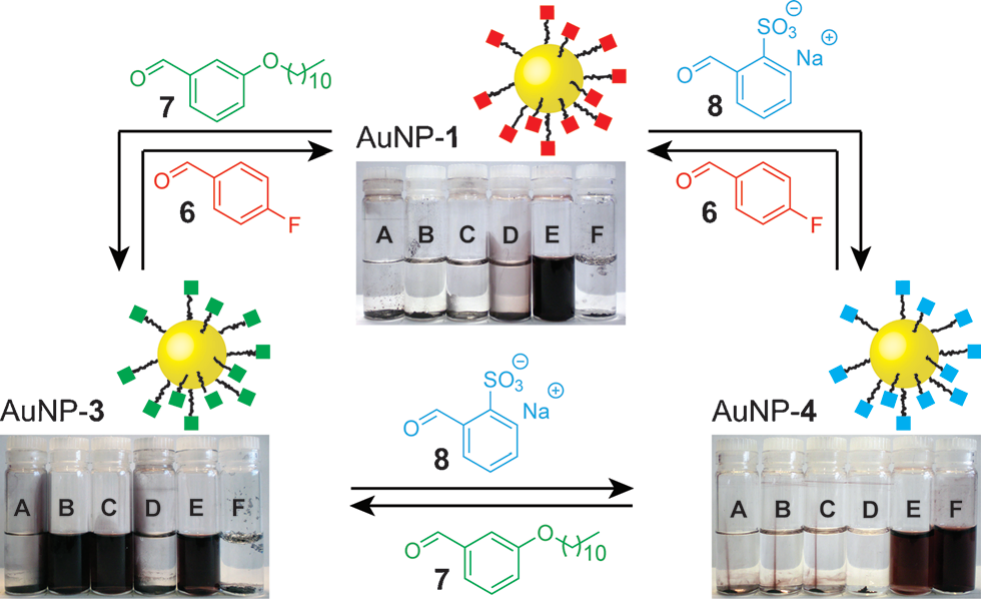
Reversible switching of AuNP solvophilicity properties by hydrazone exchange. For full conditions, see the SI, Section 11. Solvents in the inset pictures: A=hexane, B=chloroform, C=tetrahydrofuran, D=methanol, E=DMF, F=water.

In a similar manner, AuNP-**3** could be converted to AuNP-**4**, which showed excellent colloidal stability in water (Figure 4, right). *o*-Sulfonylbenzylidene hydrazone was confirmed as the major constituent of the NP-bound monolayer by a combination of ^1^H NMR spectroscopy and LDI-MS (Figures S28–S30). Each of these exchange reactions proved to be entirely reversible, such that any of the three AuNP systems, exhibiting markedly different solvophilicity properties, could be accessed from either one of the other two by treatment with the appropriate aldehyde exchange unit (Figure 4 and Scheme S2). Interestingly, during the conversion of AuNP-**3** to AuNP-**1**, a sample was obtained exhibiting solubility properties that were intermediate between the two extremes (Scheme S2). That this state arises from a mixed monolayer of hydrazones **3** and **1** was confirmed by LDI-MS analysis, which presented ion fragments originating from both possible hydrazones in roughly equal intensities (Figure S27). Subjecting this material to a further round of exchange with aldehyde **6** then yielded a sample displaying indistinguishable physical and chemical properties to AuNP-**1** produced by all other routes. Thus, it is possible to access a continuum of AuNP solvophilicity characteristics across a remarkably wide range by fine-tuning the monolayer composition through the appropriate choice of exchange conditions.

Controlling the molecular details of NP surface functionality will be critical for realizing the full technological potential of nanomaterials. Dynamic covalent NP building blocks now offer a generalizable strategy for achieving this, using simple molecular designs and mild processes that are independent of the underlying NP material. The ability to reversibly tune surface functionality raises the prospect of smart NP-based devices with environment-responsive properties, or reconfigurable self-assembly capabilities. The pseudomolecular nature of 3D NP-bound monolayers allows the direct characterization of surface-bound chemical processes, offering fundamental insights into the influence of crowded environments on reactivity, which have not been so readily accessible from analogous 2D surface-bound systems.8b Determining the complex influence of nanoscale features, such as surface curvature and monolayer composition, on reactivity is the next step that can now be addressed in the development of dynamic covalent NP building blocks to become flexible and versatile nanomaterial synthons.^25^

## Supporting Information

As a service to our authors and readers, this journal provides supporting information supplied by the authors. Such materials are peer reviewed and may be re-organized for online delivery, but are not copy-edited or typeset. Technical support issues arising from supporting information (other than missing files) should be addressed to the authors.

miscellaneous_information
